# Assessment of rice bran with acrylate or pyruvate in modulating ruminal fermentation and methane production *in vitro*

**DOI:** 10.1002/jsfa.14437

**Published:** 2025-06-13

**Authors:** Jamal James D. Manlapig, Jane Camille A. Crisostomo, Makoto Kondo, Tomomi Ban‐Tokuda, Hiroki Matsui

**Affiliations:** ^1^ Department of Animal Science College of Agriculture, Central Luzon State University Science City of Muñoz Philippines; ^2^ Graduate School of Bioresources, Mie University Tsu Japan

**Keywords:** acrylate, lactate, methane, pyruvate, rice bran

## Abstract

**BACKGROUND:**

Livestock production contributes largely to climate change and methane (CH_4_) from enteric fermentation is the primary greenhouse gas emitted from ruminants. Thus, developing effective strategies to reduce the impact of ruminant production on the environment is crucial, and the combination of CH_4_ mitigating agents may be a viable strategy to attain this. The present study aimed to evaluate the effect of rice bran alone (control+rice bran, RB) or in combination with acrylate (control+rice bran+acrylate, AA) or pyruvate (control+rice bran+pyruvate, PA) on rumen fermentation parameters, CH_4_ production, and microbial populations *in vitro.*

**RESULTS:**

Gas production was highest in control + rice bran + pyruvate (PA) treatments and lowest (*P* < 0.01) in control + rice bran + acrylate (AA), whereas dry matter digestibility (DMD) was significantly lower (*P* < 0.05) in AA compared to control (CON) and PA. CH_4_ and CH_4_/digested dry matter decreased (*P* < 0.05) by more than 66% and 63%, respectively, in AA cultures, whereas total organic acid production increased (*P* < 0.01) compared to CON. Lactate levels were significantly higher (*P* < 0.05), while acetate, propionate and butyrate were notably lower (*P* < 0.05) in AA cultures than CON. Microbial analysis showed a significant decrease in methanogen populations in AA than CON (*P* < 0.01). Compared to CON, anaerobic fungi and *Megasphaera elsdenii* populations declined across all treatments (*P* < 0.01), whereas *Selenomonas ruminantium* populations decreased in RB and AA, and *Prevotella ruminicola* populations were higher in RB and PA (*P* < 0.05).

**CONCLUSION:**

These findings suggest that incorporating RB with AA could be a viable CH_4_ inhibitor; however, because of its negative effect on gas production and DMD, further investigations should be done to determine its optimum inclusion level. © 2025 The Author(s). *Journal of the Science of Food and Agriculture* published by John Wiley & Sons Ltd on behalf of Society of Chemical Industry.

## INTRODUCTION

As concerns over climate change escalate, there is a growing urgency to develop effective strategies for mitigating methane (CH_4_) production from ruminants without compromising animal welfare or productivity.^1^ One promising research avenue involves using organic acids as dietary supplements to modulate rumen fermentation and reduce CH_4_ emissions. Organic acid supplementation has long been utilized in the livestock and poultry industries for various purposes, ranging from improving feed efficiency to enhancing animal health. These compounds, such as acetic, lactic, citric, propionic, acrylate, fumaric and malic (acids or salt‐based), exert diverse effects on animal physiology, including antimicrobial properties and modulation of rumen fermentation.^2^, ^3^, ^4^, ^5^, ^6^ Although their use in ruminant nutrition has been relatively understudied compared to monogastric animals, recent research highlights the potential of organic acids in mitigating CH_4_ emissions from ruminant livestock.^4^, ^7^

The mode of action of organic acids in ruminant nutrition involves multiple pathways contributing to CH_4_ mitigation. Organic acids, such as fumarate, malate and citrate, indirectly modulate rumen fermentation by altering substrate availability and microbial populations and promoting alternative hydrogen sinks to inhibit CH_4_ production.^3^, ^4^, ^8^, ^9^ This diversion of hydrogen away from CH_4_ formation involves the production of propionate via the succinate–propionate pathway and the acrylate pathway.^3^, ^9^ Acrylate and pyruvate, precursors of the metabolic pathways towards propionate production, also exhibit similar effects when added to rumen batch cultures, decreasing CH_4_ produced *in vitro*.^3^ However, this diversion of hydrogen towards propionate was incomplete,^10^, ^11^ yielding only less than 90% recovery,^3^ thus requiring additional additives that will complement its reported effects.

Rice bran (RB) has been reported to reduce CH_4_ production *in vitro* and *in vivo* ^12^, ^13^, ^14^ with an optimal inclusion level of 10% of the diet.^15^ Our previous results indicated that adding RB to the diet suppresses CH_4_ production directly by inhibiting the methanogen population^12^, ^16^ and indirectly by enhancing propionate production.^13^ The reduction in the methanogen population observed in treatments containing RB was attributed to compounds present in the fraction extracted using ethanol, which primarily consists of bioactive compounds. Thus, combining two different mitigating agents (rice bran and organic acids) could be an effective strategy to improve mitigation efficiency. However, very few studies were done to investigate the use of several types of mitigating agents with different types of inhibitory mechanisms, let alone their combination. Therefore, the present study aimed to assess the effects of combining rice bran with acrylate or pyruvate on *in vitro* fermentation characteristics, methanogenesis, and microbial populations.

## MATERIALS AND METHODS

### *In vitro* fermentation

Rumen fluid was obtained from three Corriedale wethers fed with oat hay and a concentrate mix (60:40) containing corn, soybean, wheat bran and alfalfa pellet meal (1:1:1:0.5). The animals were cared for following the guidelines of the Graduate School of Bioresources, Mie University, Japan. The *in vitro* fermentation system was performed following the method described in previous studies.^12^, ^13^, ^16^ Ruminal fluid was collected using an oral stomach tube before the morning feeding and immediately transported to the laboratory. The ruminal fluid was strained through four layers of gauze and pooled together in equal proportions. Pooled ruminal fluid was mixed with McDougall buffer (1:4) along with continuous purging with nitrogen (N_2_) gas. The buffered ruminal fluid was maintained at 39 °C in a water bath and used as inoculum in *in vitro* incubations.

The *in vitro* incubations were performed using 125‐mL bottles containing approximately 600 mg of substrates and 29 mL of buffered rumen fluid. For the control (CON), 210 mg of oat hay, 210 mg of corn starch and 180 mg of wheat bran were used as substrates. The treatments contain similar amounts of oat hay and cornstarch, and 30% of the wheat bran (10% of the total substrate) was replaced by rice bran (120 mg of wheat bran and 60 mg of rice bran). On the day of incubation, bottles containing substrate were gassed with N_2_ before adding 29 mL of buffered rumen fluid plus 1 mL of distilled water for CON and treatment 1 (control + rice bran, RB). Sodium acrylate and sodium pyruvate (sodium salt; FUJIFILM Wako Pure Chemical Corporation, Tokyo, Japan) were dissolved in distilled water (0.6 m) and 1 mL of the corresponding solution was added to the bottles containing 29 mL of buffered rumen fluid before incubation. The final concentration for acrylate (control + rice bran + acrylate, AA) and pyruvate (control + rice bran + pyruvate, PA) treatments was 20 mmol mL^−1^. The bottles were fitted with butyl rubber stoppers, capped with aluminum crimped seals to prevent the escape of fermentation gases, and incubated for 24 h at 39 °C in a shaking water bath at 170 oscillations per minute. Each treatment was replicated three times and incubated on three separate days.

### Analysis of gas and fermentation end products

After a 24‐h incubation period, the amount of gas generated in the cultures was measured with a glass syringe equipped with a Luer‐lock three‐way stopcock and a 21‐gauge needle. The bottles were immediately placed in ice to stop fermentation and then processed. A gas chromatography system (CG8AIT; Shimadzu Corporation, Kyoto, Japan) fitted with a Shincarbon ST column (diameter, 3 mm; length, 2 m) and a thermal conductivity detector was used to examine the composition of headspace gas. The temperature settings for the column and detector are 100 and 150 °C, respectively. High‐purity argon gas (99.999%) was employed as the carrier gas and this was used at a rate of 50 mL min^−1^. Gas composition and gas concentration (%) were calculated using an integrator (C‐R8A; Shimadzu Corporation). CH_4_ in gas was calculated as described by Lopez and Newbold.^17^ A pH meter (Horiba F‐52; Horiba Advanced Techno, Co., Ltd, Kyoto, Japan) was used to measure the pH. The residual substrate was collected from the remaining supernatant by centrifugation at 1000 × *g* for 10 min at 4 °C and used to determine dry matter digestibility (DMD). Following centrifugation, the pellet was transferred to pre‐weighed aluminum containers and placed in an oven dryer for 48 h at 80 °C. Subsequently, the residues were weighed before (0 h) and after (24 h) incubation, and the mass loss as opposed to the pre‐digestion mass was estimated using: $\mathrm{DMD}\%=\left[\frac{0\;h-24\;h}{0\;h}\right]\times 100$. The quantity of CH_4_ produced per gram of digested dry matter (DDM) was calculated by dividing the mass of DDM by the amount of CH_4_ produced: $\frac{CH_{4}}{\mathrm{DDM}}=\frac{C{H_{4}}_{\text{produced}}}{\mathrm{DD}M_{\left\{0\;h\right\}}–\mathrm{DD}M_{\left\{24\;h\right\}}}$ Using a HPLC system (Shimadzu Corporation) consisting of a Shimadzu LC‐20 AD pump, a liquid chromatography SIL‐20A autosampler and a CDD‐20A conductivity detector, the concentrations of organic acid were determined using a modified method by Manlapig *et al*.^13^ The analytes were separated on two ICSep COREGEL‐87H3 columns (7.8 mm inner diameter × 300 mm length) at 45 °C. The flow rate was 0.6 mL min^−1^ and the injection volume was 20 μL. Standard solutions of lactate, acetate, propionate, butyrate, acrylate, pyruvate and succinate were utilized for the analysis following the appropriate dilutions (0.05–50 mmol).

### DNA extraction and real‐time PCR analysis

The microbial DNA was extracted using a NucleoSpin DNA Stool Mini Kit (Macherey‐Nagel GmbH & Co., Düren, Germany) in accordance with the manufacturer's instructions with some modifications.^18^ The microbial cells were disrupted with a ShakeMan6 homogenizer (Bio‐Medical Science, Tokyo, Japan) and the eluted DNA was then stored at −80 °C until use.

A real‐time PCR assay was performed using Thunderbird® SYBR® qPCR Mix (TOYOBO, Osaka, Japan) and a StepOne Plus™ Real‐Time PCR System (Applied Biosystems, Foster City, CA, USA). The PCR reaction mixture (20 μL) contained 10 μL of Thunderbird SYBR qPCR mix (TOYOBO), 0.4 μL of ROX dye, specific forward and reverse primers, 1 μL of DNA template and sterile Milli‐Q water (Merck Millipore, Burlington, MA, USA). The following conditions were used for the PCR cycle: an initial denaturation step at 95 °C for 1 min for initial denaturation, followed by 40 cycles of denaturation at 95 °C for 15 s, with annealing at the temperatures shown in Table 1 for 30 to 60 s except for protozoa, *Prevotella ruminicola* and *Megasphaera elsdenii*, which include extension at 72 °C. Amplicon specificity was determined via dissociation curve analysis of PCR end products by increasing the temperature at a rate of 1°C 30 s^−1^ from 60 °C to 95 °C. The calculation was performed using StepOne Software, version 2.2 (Applied Biosystems).

**Table 1:** Primers used for the analysis of the microbial population using real‐time PCR

Target	Sequence(5′‐ to 3′)	Annealing temperature (°C)	References
Total bacteria	CGGCAACGAGCGCAACCC	60	43
	CCATTGTAGCACGTGTGTAGCC		
Protozoa	GCTTTCGWTGGTAGTGTATT	55	44
	CTTGCCCTCYAATCGTWCT		
Anaerobic fungi	GAGGAAGTAAAAGTCGTAACAAGGTTTC	60	43
	CAAATTCACAAAGGGTAGGATGATT		
Methanogen	TTCGGTGGATCDCARAGRGC	60	45
	GBARGTCGWAWCCGTAGAATCC		
*Selenomas ruminantium*	CAATAAGCATTCCGCCTGGG	60	46
	TTCACTCAATGTCAAGCCCTGG		
*Butyrivibrio fibrisolvens*	ACCGCATAAGCGCACGGA	60	47
	CGGGTCCATCTTGTACCGATAAAT		
*Megasphaera elsdenii*	GACCGAAACTGCGATGCTAGA	58	48
	CGCCTCAGCGTCAGTTGTC		
*Prevotella ruminicola*	GGTTATCTTGAGTGAGTT	53	34
	CTGATGGCAACTAAAGAA		

### Statistical analysis

*In vitro* gas production and composition, DMD, organic acids, CH_4_, and microbial population data were analyzed using a completely randomized design by one‐way analysis of variance in SAS, version 9.4 (SAS Institute Inc., Cary, NC, USA) using the model: *Y*_ij_ = *μ* + *A*_i_ + *e*_ij_, where *Y*_ij_ is the observation, *μ* is the overall mean, *A*_i_ is the treatment and *e*_ij_ is the error term. All data are presented as the mean ± SEM. *P* < 0.05 was considered statistically significant. Tukey's multiple range test was used to identify differences between treatments.

## RESULTS

The data on pH, gas production, DMD and CH_4_ production are presented in Table 2. After 24 h of incubation, gas production and DMD in cultures containing AA were significantly lower than in CON, RB and PA (*P* < 0.05). CH_4_ (%), CH_4_ (mL) and CH_4_/DDM significantly decreased in response to the addition of AA in cultures compared to CON and other treatments (*P* < 0.05).

**Table 2:** Fermentation characteristics and methane production after 24 h of *in vitro* incubation

	CON	RB	AA	PA	SEM	*P*‐value
pH						
0 h	7.94	8.03	8.04	8.07	0.07	0.994
24 h	6.23	6.31	6.10	6.34	0.04	0.172
Gas (mL)	101.33 ^a^	99.56 ^a^	76.44 ^b^	103.33 ^a^	3.41	<0.0001
DMD (%)	51.32 ^ab^	51.61 ^a^	48.52 ^b^	52.49 ^a^	1.00	0.016
CH_4_ (%)	13.45 ^a^	11.13 ^ab^	6.18 ^b^	11.18 ^ab^	1.16	0.033
CH_4_ (mL)	13.58 ^a^	11.07 ^ab^	4.78 ^b^	11.62 ^a^	0.53	0.011
CH_4/_DDM (mL g^−1^)	51.76 ^a^	42.18 ^ab^	19.43 ^b^	43.59 ^ab^	4.29	0.015

Abbreviations: AA, control + rice bran + acrylate (20 mmol L^−1^); CH_4_, methane; CH_4_/DDM, methane per digested dry matter; CON, control; DMD, dry matter digestibility; PA, control + rice bran + pyruvate (20 mmol L^−1^); RB, control + rice bran. Superscripts with different letters in a row are significantly different. SEM, standard error of the mean.

Total and individual organic acid production are presented in Table 3. Total organic acid was highest in AA, followed by PA, CON and RB. The addition of AA and PA significantly increased (*P* < 0.01) the production of organic acid after 24 h of incubation compared to CON. The lactate produced was significantly higher in AA than in other treatments (*P* < 0.01). Cultures containing PA also have higher (*P* < 0.05) lactate than CON and RB. Conversely, acetate production after 24 h significantly decreased in response to the addition of AA in cultures (*P* < 0.01). Similarly, propionate and butyrate produced in AA were significantly lower than CON, RB and PA (*P* < 0.05). Propionate production reduced significantly (*P* < 0.01) with the addition of organic acids (AA and PA) in cultures, whereas butyrate only decreased in AA (*P* < 0.05) and increased in response to the addition of PA compared to CON (*P* < 0.05). Acrylate and succinate were only detected in treatments containing AA.

**Table 3:** Organic acid production (mmol L^−1^) after 24 h of *in vitro* incubation

	CON	RB	AA	PA	SEM	*P*‐value
Total OAs	95.70 ^c^	91.08 ^d^	112.69 ^a^	102.76 ^b^	8.70	<0.0001
Lactate	0.02 ^c^	0.14 ^bc^	53.33 ^a^	11.24 ^b^	2.93	<0.0001
Acetate	52.57 ^a^	46.70 ^bc^	29.36 ^c^	53.61 ^a^	1.68	<0.0001
Propionate	29.80 ^a^	28.41 ^a^	15.75 ^c^	22.74 ^b^	0.99	<0.0001
Butyrate	13.30 ^b^	15.83 ^a^	7.62 ^c^	15.17 ^a^	0.60	<0.0001
Acrylate	ND	ND	2.38	ND	0.07	
Succinate	ND	ND	4.25	ND	2.50	

Abbreviations: AA, control + rice bran + acrylate (20 mmol L^−1^); CON, control; ND, not detected; OA, organic acid; PA, control + rice bran + pyruvate (20 mmol L^−1^); B, control + rice bran. Superscripts with different letters in a row are significantly different. SEM, standard error of the mean.

**Figure 1:**
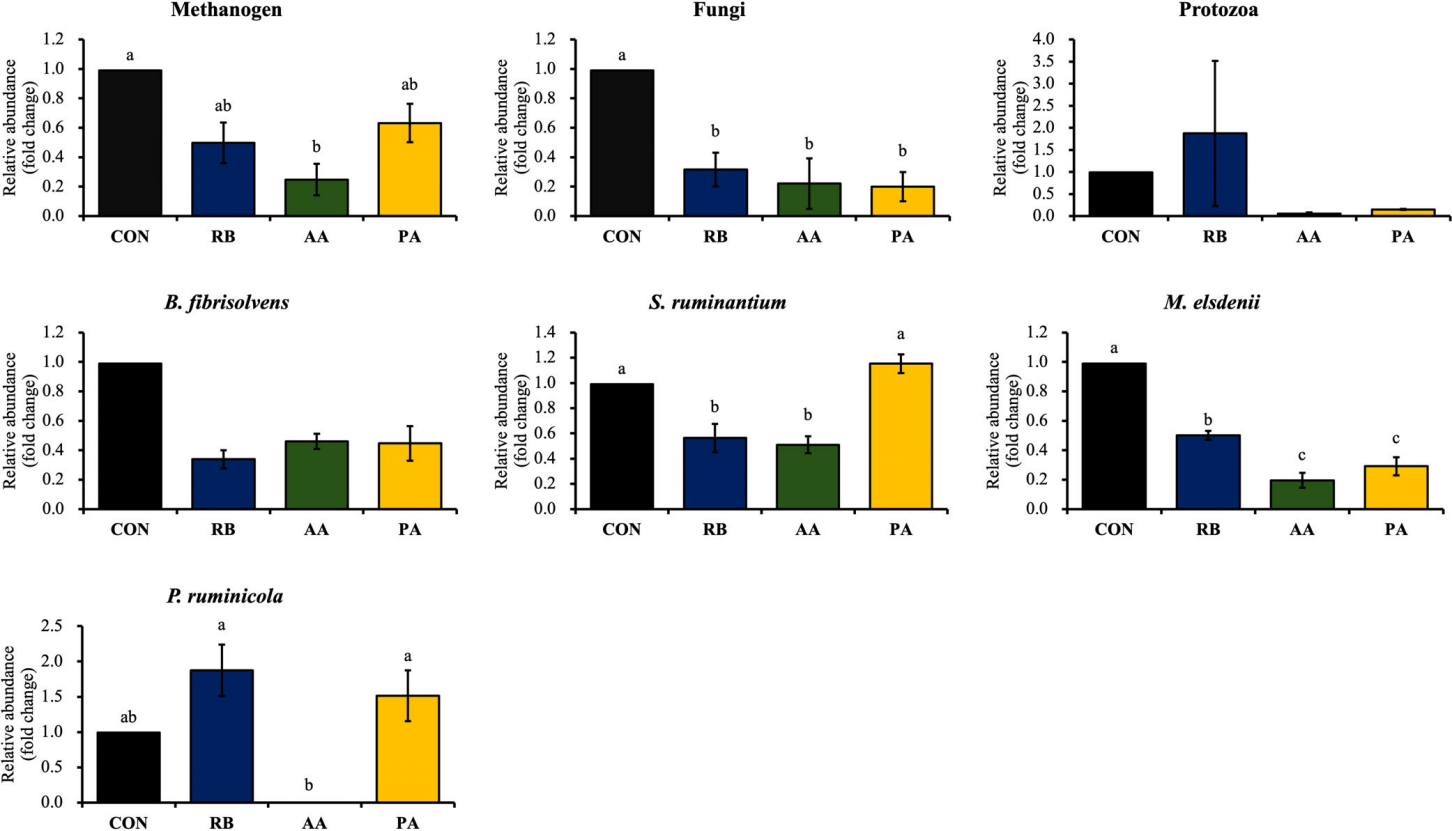
Effect of rice bran and organic acid addition on microbial populations *in vitro* determined by real‐time PCR. Data were presented as the mean ± SEM (*n* = 3). ^a,b,c^ Means with different superscripts differ significantly (*P* < 0.01). CON, control; RB, control + rice bran; AA, control + rice bran + acrylate; PA, control + rice bran + pyruvate.

The microbial population of cultures after 24 h of *in vitro* fermentation as influenced by the addition of rice bran with or without AA or PA is presented in Fig. 1. The methanogen population significantly decreased in AA compared to CON (*P* < 0.01). The populations of anaerobic fungi and *M. elsdenii* were significantly decreased (*P* < 0.01) in RB, AA, and PA compared to CON. Compared to CON, the population of *Selenomonas ruminantium* decreased (*P* < 0.05) in RB and AA. A higher population of *P. ruminicola* was observed with RB and PA than in AA (*P* < 0.05), but it was comparable to CON.

## DISCUSSION

Organic acid supplementation in ruminants has been shown to influence fermentation characteristics, total organic acid levels, CH_4_ production and microbial populations.^3^, ^4^, ^7^, ^19^ In the present study, the supplementation of AA and PA in cultures did not affect the pH before and after incubation, in contrast to the existing literature suggesting that adding organic acid to cultures typically results in pH changes after fermentation.^3^, ^20^ This change is usually linked with the uptake and utilization of lactate by microorganisms in the rumen.^19^ However, this was not observed in the present study, possibly because the organic acids used were calcium salts and the accumulation of lactate in the treatments containing AA and PA. After 24 h of incubation, the pH values of all cultures were not significantly different, ranging from 6.10 to 6.34. One limitation of the present study is the lack of time‐series data for pH measurements, which may have overlooked transient but significant changes occurring between 0 and 24 h. Kolver and De Veth^21^ suggested that the ruminal pH decreased with the concentration of volatile fatty acids (VFA). Judging from this fact, the pH of the culture fluid decreased with organic acid production in the present study. Among the treatments, cultures containing AA had the lowest pH. Although statistically similar to the CON and other treatments, this result may have contributed to the observed reduction in gas production and DMD in the culture with AA. It is well established that lower pH decreases substrate degradability through reduced fiber degradation,^22^, ^23^ and *in vitro* gas production is positively correlated to DMD.^24^ In cows consuming a mixed diet, researchers reported that rumen fluid pH below 6.2 would cause a moderate reduction in fiber digestion^25^ by lowering the activity of fibrolytic microorganisms that degrade cell walls.^26^, ^27^ Moreover, Lopez‐Aguirre *et al*.^28^ observed that fiber degradability in the diet directly impacts DMD and reported that the addition of 1 μL per 0.5 g DM of cellulase in an *in vitro* system improved DMD and increased total gas production. In the present study, the gas production in AA was 24.5%, 23.2% and 26% lower than CON, RB and PA treatments, respectively. Additionally, DMD was also reduced by at least 5% in AA compared to other treatments. These results suggest that adding AA in cultures may negatively affect fermentation characteristics and limit its practical applicability in ruminant diets.

**Figure 2:**
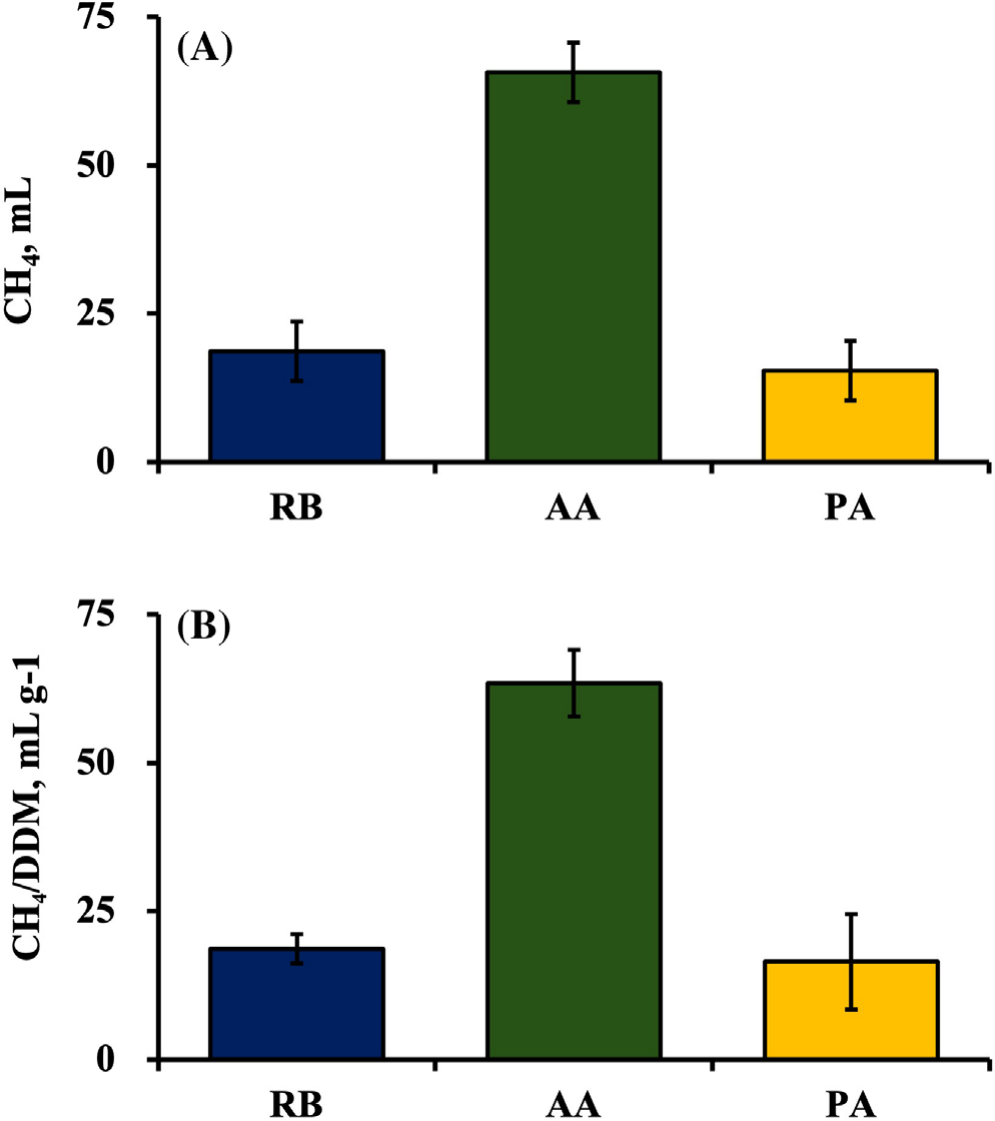
Reduction in (A) methane (CH_4_) and (B) methane per digested dry matter (CH_4_/DDM) in treatments as opposed to the control after 24 h of incubation. Data are presented as the mean ± SEM (*n* = 3). RB, control + rice bran; AA, control + rice bran + acrylate; PA, control + rice bran + pyruvate.

Inhibition of CH_4_ production has been reported in diets containing rice bran^12^, ^13^, ^16^ and organic acids.^3^, ^4^, ^10^ In the present study, rice bran was used in combination with acrylate or pyruvate to determine whether there was an additive or synergistic effect on their CH_4_ suppressing potential. Expectedly, CH_4_ concentration (%), CH_4_ production (mL) and CH_4_/DDM (mL g^−1^) were decreased in all treatments. However, the reduction observed in RB and PA (11.13% *vs*. 11.18% and 11.07 mL *vs*. 11.62 mL, respectively) was almost identical, indicating that adding PA in cultures containing rice bran does not enhance CH_4_ inhibition. Conversely, AA yielded greater reductions compared to other treatments, achieving 54% and 64% reductions for CH_4_ production and CH_4_/DDM (Fig. 2), respectively, compared to CON. Generally, organic acid supplementation inhibits CH_4_ production indirectly by diverting reducing equivalents from ruminal methanogenesis to alternative electron sinks, such as the production of propionate.^29^ Malate, fumarate, lactate and acrylate accept one pair of electrons in their conversion into propionate.^3^, ^30^, ^31^ However, the propionate produced in cultures containing PA and AA was lower compared to CON. The reduction in CH_4_ produced in cultures containing AA was greater than that in PA, and, after the gas composition analysis, hydrogen was only detected in cultures containing AA (data not presented). These findings indicate that the observed reduction in CH_4_ production in AA cultures was not a result of the utilization of hydrogen toward propionate production, suggesting that an alternative mechanism may be involved.

It is thus apparent that RB and organic acid can alter ruminal fermentation characteristics. In this study, the addition of RB in the cultures reduced the total organic acids produced compared to CON, similar to the findings reported by Manlapig *et al*.^13^ By contrast, adding AA and PA increased organic acid production in the cultures. Newbold *et al*.^3^ reported that the addition of pyruvate and acrylate (in a high forage diet) increased the total VFA produced after 24 h of incubation. Similar results were also reported for other organic acids such as fumarate,^3^, ^7^ citrate,^4^, ^32^ itaconate and maleic anhydride.^4^ However, the composition of organic acids produced in AA and PA differed from each other. Lactate accumulated in both treatments; however, the amount in AA was almost five times that in PA. Consequently, the amount of acetate, propionate and butyrate was higher in PA than in AA. This suggests that, although both AA and PA resulted in higher organic acid levels, their metabolisms and impact on rumen fermentation differ. Pyruvate can be converted into acetate, producing 2H and/or lactate via the acrylate pathway, utilizing 2H during the process.^3^ These pathways may further explain the lack of observed reduction in CH_4_ in cultures containing PA, where higher acetate and lower lactate were produced after incubation, suggesting that relatively higher H_2_ was available for CH_4_ production.

Although the effect of several organic acids on rumen fermentation has been reported numerous times, limited information is available on their impact on microbial populations. Yamada *et al*.^4^ reported that the methanogenic archaea were not greatly affected by the supplementation of organic acids according to generic classifications. Similarly, methanogen populations were not affected by the addition of pyruvate in the cultures. It has been reported previously that rice bran can reduce the methanogen population *in vitro*.^12^, ^16^ In the present study, treatments containing rice bran have numerically lower populations of methanogens compared to CON. However, significant reductions were only observed between CON and AA after 24 h of incubation, indicating that AA addition in rice bran diets may add to its anti‐methanogenic effect. This effect was also observed in diets containing rice bran and fumarate.^7^

The anaerobic fungal population was decreased in response to the treatment (RB, AA and PA) inclusion. However, based on the comparison of their relative abundance, no differences were found between the treatments. This suggests that the decrease observed in treatments was the result of rice bran addition in the diets rather than organic acids (AA and PA). Similar results were reported in diets with rice bran, decreasing the amounts of fibrolytic bacteria (anaerobic fungi, *Fibrobacter succinogenes* and *Ruminococcus albus*) after 24 h of incubation.^13^ Replacing wheat bran with rice bran in the treatments reduced fibrous carbohydrates in the diet. This dietary shift not only affects the composition of the feed, but also influences the rumen microbial environment. Studies by Mosoni *et al*.^33^ and Tajima *et al*.^34^ provide evidence that a decrease in fibrous and an increase in readily fermentable carbohydrates are associated with a decline in the population of fiber‐degrading bacteria.

In the rumen, key lactate‐utilizing bacteria such as *S. ruminantium* and *M. elsdenii* play a crucial role in breaking down lactate through lactate dehydrogenase, ultimately converting it into VFAs.^35^, ^36^ Our study employed real‐time PCR and found that the addition of AA led to a decrease in the population of *S. ruminantium*. A similar reduction in *M. elsdenii* was noted with both AA and PA. *S. ruminantium* transforms lactate into propionate via the succinate pathway through decarboxylation of succinate,^37^, ^38^ whereas *M. elsdenii* employs the acrylate pathway to convert lactate into propionate.^38^ Moreover, *M. elsdenii* can also produce butyrate from lactate.^38^ The decline in these bacterial populations may contribute to the observed accumulation of lactate and the corresponding decrease in VFA production following treatments with AA and PA. Our findings indicate that high lactate accumulation could negatively impact VFA production. Nonetheless, further investigation is necessary to fully understand how AA influences the populations of *S. ruminantium* and *M. elsdenii*.

The genus *Prevotella* of the phylum *Bacteroidetes* (now *Bacteroidota*) is one of the most predominant genera in the rumen,^39^ and it is involved in the metabolism of carbohydrates, which are converted into propionate and acetate. In the present study, the relative abundance of the *Prevotella ruminicola* population in AA was decreased (0.01‐fold change) and lower than in PA (1.5‐fold change), thus further explaining the differences in VFA and CH_4_ produced in the cultures. Aguilar‐Marin *et al*.^40^ reported that lower CH_4_ emissions were associated with a higher abundance of ruminal *Prevotella* in a cohort of Colombian buffalos. Similarly, Shinkai *et al*.^41^ reported that high populations of *Prevotella* in the rumen may be associated with low CH_4_ and high propionate production, in contrast to the results obtained in the present study. However, Danielsson *et al*.^42^ reported that a higher abundance of OTUs of *Prevotella* was detected in both high and low CH_4_ phenotypes. These mixed results suggest that CH_4_ production and *Prevotella* population may have a complex relationship, likely as a result of the differences in substrates and fermentation end products.

## CONCLUSIONS

The present study highlights the effects of acrylate and pyruvate supplementation on ruminal fermentation characteristics, microbial populations and methane production. Although the addition of acrylate reduced gas production, dry matter digestibility and population of lactate‐utilizing microorganisms, it also led to increased total organic production and significant reductions in methane output and methanogen populations, which is promising for efforts to mitigate greenhouse gas emissions from livestock. Further research is needed to identify the optimal inclusion level of acrylate that strikes an ideal balance between enhancing diet digestibility and reducing methane emissions and elucidate the mechanisms by which acrylate influences microbial populations and fermentation efficiency, which will be crucial for its dietary application on ruminant diets.

## CONFLICTS OF INTEREST

The authors declare that they have no conflicts of interest.

## Data Availability

The data that support the findings of this study are openly available in Mie University Scholarly E‐collections at https://mie‐u.repo.nii.ac.jp/.
